# Comorbidities in patients with chronic obstructive pulmonary disease: a comprehensive study

**DOI:** 10.25122/jml-2022-0057

**Published:** 2023-07

**Authors:** Nagham Yahya Ghafil, Falah Mahdi Dananah, Ekhlas Sabah Hassan, Yarob Saad Abdiljaleel Alkaabi

**Affiliations:** 1 Department of Pharmacology and Toxicology, Faculty of Pharmacy, University of Kufa, Kufa, Iraq; 2 Department of Physiology, Faculty of Medicine, University of Kufa, Kufa, Iraq; 3 Department of Pharmacology and Therapeutics, Faculty of Medicine, University of Kufa, Kufa, Iraq

**Keywords:** type 2 diabetes mellitus, atherosclerotic cardiovascular diseases, hypertension, dyslipidemia, COPD

## Abstract

In recent years, there has been an increasing interest in understanding the systemic nature of COPD and its frequently associated comorbidities. COPD is characterized by chronic lung disease involving local and systemic inflammation and non-reversible airway obstruction. The disease course is marked by recurrent exacerbations and is often accompanied by various comorbidities. This study aimed to evaluate the prevalence of comorbidities among Iraqi patients with COPD and their association with disease severity. A case-control study was conducted at Al-Sader Hospital in Annajaf from October 2019 to October 2020, involving 200 participants. The study population comprised 100 patients with COPD (COPD group) and 100 individuals without COPD serving as the control group. Patients with COPD were divided into four groups according to the disease severity. The prevalence of type 2 diabetes mellitus (T2DM), atherosclerotic cardiovascular diseases (ASCVD), hypertension, and dyslipidemia was determined in all groups. Patients with COPD had a significantly higher prevalence of T2DM, ASCVD, hypertension, and dyslipidemia, and, except for T2DM, the prevalence was significantly higher in the more severe groups. It was concluded that T2DM, ASCVD, hypertension, and dyslipidemia were commonly associated with COPD.

## INTRODUCTION

Chronic Obstructive Pulmonary Disease (COPD) refers to a pulmonary disease that is chronic and highly preventable but only partially treatable with associated airway and systemic inflammation.

Patients with COPD usually experience recurrent exacerbations over its course, which may require hospitalization. Additionally, COPD is associated with various manifestations, such as anorexia, poor nutrition, weight loss, and impaired skeletal muscle function, which can be attributed to underlying systemic inflammation [1, 2]. On a global scale, COPD has been and is estimated to continue to be one of the major health issues consuming a considerable share of global health resources. In 2015, COPD accounted for 5% of deaths worldwide, with the majority occurring in low- and middle-income regions. The burden of COPD is projected to increase, making it the third leading cause of death globally by 2030 [3]. Comorbidities are common in COPD patients, and understanding their frequency, nature, severity, and impact has been the subject of extensive research. Various theories have emerged to explain the relationship between these comorbidities and COPD.

Systemic inflammation, a hallmark of COPD, is believed to play a role in the development of comorbidities. This inflammation may result from the airway inflammation seen in COPD and subsequent activation of systemic inflammatory cascades. Another explanation is that the pulmonary abnormalities in COPD could be a manifestation of a broader inflammatory process involving multiple body systems [4]. Patients with chronic respiratory diseases, including COPD, have higher mortality rates following myocardial infarction compared to other groups [5].

Type 2 diabetes mellitus (T2DM) has a higher prevalence in COPD patients compared to the general population, independent of age, body mass index, gender, or smoking status [6]. Patients with COPD also have a higher prevalence of different types of dyslipidemia even after adjudgment for smoking [7, 8]. Studies have suggested that inflammatory cytokines and mediators present in COPD patients may contribute to the development of cardiovascular comorbidities [9]. This study aimed to identify the prevalent comorbidities in a sample of Iraqi patients with COPD.

## MATERIAL AND METHODS

This study was conducted at Al-Sader Hospital in Najaf, Iraq, from October 2019 to October 2020. The study included a total of 200 participants who were divided into two groups:

1. The COPD group consisted of 100 patients diagnosed with COPD based on clinical symptoms and Spirometric tests.
2. Control group: This group comprised 100 randomly selected subjects confirmed to be free from COPD based on appropriate clinical and Spirometric evaluations.

Within the COPD group, patients were further categorized into four subgroups based on the severity of their disease, following the criteria set by the Global Initiative for Chronic Obstructive Lung Disease (GOLD) [1]:

- Mild COPD: FEV1/FVC<70%, FEV1≥80% predicted (n=32).
- Moderate COPD: FEV1/FVC<70%, 50%≤FEV1<80% predicted (n=34).
- Severe COPD: FEV1/FVC<70%, 30%≤ FEV1< 50% predicted (n=22).
- Very severe COPD: FEV1/FVC<70%, FEV1<30% predicted (n=12).

The following definitions were used for the assessment of diseases and comorbidities:

1. COPD: Presence of dyspnea, cough, sputum production, and exposure to relevant risk factors, along with a positive Spirometric test (FEV1/FVC%< 70) [10].
2. Atherosclerotic cardiovascular diseases: History of myocardial infarction, stroke, congestive heart failure, or angina pectoris [7].
3. Hypertension: Diagnosis and/or treatment of hypertension or evidence of elevated blood pressure (diastolic pressure≥90 mmHg or systolic pressure≥140 mmHg) [11].
4. Type 2 diabetes mellitus: Fasting blood glucose>126 mg/dL or current treatment for diabetes [11].
5. Dyslipidemia: Presence of one or more of the following criteria [11]:
  - Hypercholesterolemia: Fasting total cholesterol>240 mg/dL or using cholesterol-lowering medication.
  - Hypertriglyceridemia: Fasting serum triglyceride>200 mg/dL.
  - Low HDL cholesterol: Fasting serum HDL cholesterol<40 mg/dL.

Lung function was assessed using Spirolab III MIR^®^, Italy, following accepted criteria [12]. Participation in the study was voluntary and informed written consent was obtained from all participants. The acquired data were analyzed using the chi-square test and cross-sectional data analysis, with a significance level set at p<0.05 [13].

## RESULTS

Two hundred subjects were enrolled in the study, including 100 patients with COPD (COPD group) and 100 subjects without COPD (control group). Table 1 presents the key characteristics of the two groups.

**Table 1:** Characteristics of study groups - gender, age, BMI, and smoking history

	Gender		Age (year)	BMI	Smoking (Pack-years)
	male	female			
**COPD**	85	15	52.64 (±9.34)	21.72(±3.29)	48.60(±26.50)
**CONTROL**	85	15	51.89(±10.23)	22.3(±2.40)	50.1(±21.44)

**Figure 1:**
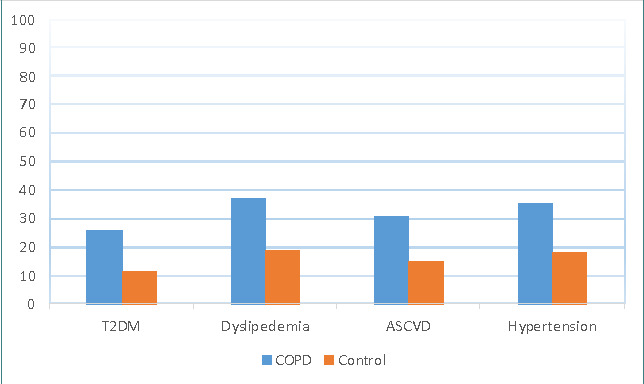
Prevalence of T2DM, dyslipidemia, ASCVD, and hypertension among patients with COPD and the control group

**Figure 2:**
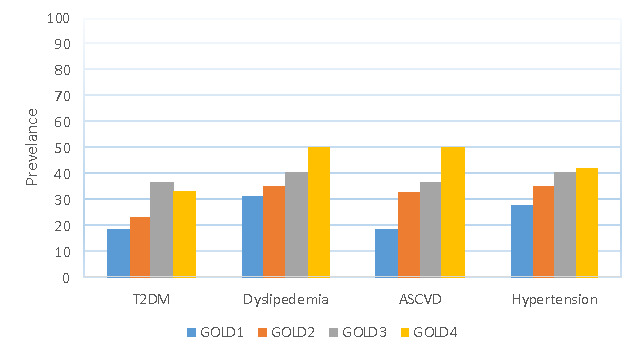
Prevalence of T2DM, dyslipidemia, ASCVD, and hypertension among patients with different GOLD stages of COPD

The two study groups showed no significant differences in mean age, gender distribution, body mass index, and smoking status. The results show that 39% of the participants had one or more comorbidities investigated in the study. Among the COPD patients, 55% had one or more comorbidities, while 33% of the control group had at least one comorbidity. Figure 1 illustrates the prevalence of T2DM, ASCVD, dyslipidemia, and hypertension in the COPD and control groups, while Figure 2 shows the prevalence of these comorbidities among patients with different GOLD stages of COPD.

The prevalence of T2DM in COPD patients was 26% compared to 11% in the control group (OR 2.84, 95% CI 1.32, 6.14). Fisher’s exact test demonstrated a significant difference in T2DM between COPD and the control subjects (26% *vs*. 11%, p=0.01) (Figure 1). Among COPD patients, the highest prevalence of T2DM was in the GOLD 3 (severe) group (Figure 2).

Dyslipidemia was significantly more prevalent in patients with COPD (37%) compared to the control group (19%), as indicated by Fisher's exact test (p=0.007) (Figure 1). The unadjusted odds ratio for dyslipidemia associated with COPD was 2.5 (95% CI 1.3, 4.8). Figure 2 illustrates the prevalence of dyslipidemia among patients with different COPD stages, with the highest prevalence in COPD patients with GOLD 4. Atherosclerotic cardiovascular disease (ASCVD) was present in 15% of the control subjects versus 31% in patients with COPD (Figure 1). This difference in prevalence was statically significant (p<0.05) using Fisher’s exact test (OR 2.6, 95% CI 1.3, 5.1). Patients with GOLD stage 4 had a higher prevalence of CVDs than patients with other GOLD stages, as shown in Figure 2.

Hypertension was commonly associated with COPD patients (35%), in contrast to 18% of the control group (Figure 1). This difference was statistically significant (p<0.05) based on Fisher's exact test, and the odds ratio for this association was 2.5 (95% CI 1.3, 4.7). Patients with GOLD stage 4 demonstrated a prevalence of 42%, which was higher than other groups (Figure 2).

## DISCUSSION

The findings of this study indicate a higher prevalence of comorbidities in COPD patients, with variations observed based on the severity of COPD. Patients with COPD had a higher prevalence of T2DM, hypertension, atherosclerotic cardiovascular diseases, and dyslipidemia than the control group. While data on the epidemiology of COPD and its comorbidities in Iraq is limited, some studies have reported increased gastroesophageal reflux symptoms among Iraqi COPD patients, with no association found with thyroid dysfunction or fibromyalgia [14-16].

Studies investigating comorbidities in COPD patients worldwide are becoming more common, emphasizing the potential shared pathophysiological mechanisms between COPD and various comorbidities [17-20]. Our findings are generally consistent with the results of these international studies. Divo *et al*. suggested that comorbidities are important in the prognosis and morbidity of COPD [17]. Corlateanu *et al*. estimated that comorbidities are detected in up to 80% of COPD patients and that, similar to the results obtained in our study, the prevalence of diabetes mellitus, hypertension, and elevated lipids in the blood increases as the disease stage of COPD increases in severity [22].

The association between respiratory and cardiovascular diseases, including COPD and hypertension, can be attributed to systemic inflammation, chronic infections, shared risk factors, and endothelial dysfunction [7]. The reduced vascular elasticity observed in COPD patients, regardless of disease severity, may contribute to the increased association with hypertension [8]. Furthermore, endothelial dysfunction may be the result of reduced circulating endothelial progenitor cells, which are responsible for maintaining endothelial integrity and enhancing the repair process following injury [21, 22].

The higher prevalence of T2DM in COPD patients, even in mild cases and independent of steroid use, may be attributed to elevated levels of inflammatory mediators such as TNF-a and IL-6, which can lead to insulin resistance [23]. The derangements in lipid profiles observed in COPD patients are consistent with previous studies that have explored the impact of airflow obstruction in COPD on lipid profiles and subsequent cardiovascular morbidity and mortality [24, 25], although some researchers reported contradicting results [26]. These derangements can be partly attributed to the complex interplay between various lipid components and the chronic elevation of inflammatory cytokines in COPD.

## CONCLUSION

Our study highlights the common comorbidities such as type 2 diabetes mellitus, atherosclerotic cardiovascular diseases, hypertension, and dyslipidemia in patients with COPD. Importantly, the prevalence of these comorbidities increased with the severity of COPD, particularly in patients with severe and very severe disease.
